# Comparative study on bone mineral density in premenopausal patients with estrogen receptor-positive breast cancer in ASTRRA Study: a 5-year follow-up study

**DOI:** 10.3389/fonc.2025.1465256

**Published:** 2025-10-02

**Authors:** Eunju Shin, Seung Il Kim, Min-ho Park, Hyun-Ah Kim, Yongsik Jung, Jai Min Ryu, Eun Hwa Park, Sung Yong Kim, Eun-Gyeong Lee, Min Hyuk Lee, Jung Ho Park, Seock-Ah Im, Soong June Bae, Su Hwan Kang, Woo Sung Lim, Hyun Jo Youn, Heung Kyu Park, Kyong Hwa Park, Tae Hyun Kim, Shin Young Park, Cheol Wan Lim, Geum Hee Kwak, Chanheun Park, Hyuk Jae Shin, Young Bum Yoo, Sun Hee Kang, Bong Kyun Kim, Hee Jeong Kim

**Affiliations:** 1 Division of Breast Surgery, Department of Surgery, University of Ulsan College of Medicine, Asan Medical Center, Seoul, Republic of Korea; 2 Department of Surgery, Kyung Hee University Hospital at Gangdong, Seoul, Republic of Korea; 3 Department of Surgery, Yonsei University College of Medicine, Seoul, Republic of Korea; 4 Department of Surgery, Chonnam National University Medical School, Chonnam National University Hwasun Hospital, Jeollanam-do, Republic of Korea; 5 Department of Surgery, Korea Cancer Center Hospital, Korea Institute of Radiological and Medical Sciences, Seoul, Republic of Korea; 6 Department of Surgery, Ajou University School of Medicine, Suwon, Republic of Korea; 7 Division of Breast Surgery, Department of Surgery, Samsung Medical Center, Sungkyunkwan University School of Medicine, Seoul, Republic of Korea; 8 Department of Surgery, Dong-A University College of Medicine, Busan, Republic of Korea; 9 Department of Surgery, Soonchunhyang University, College of Medicine, Cheonan Hospital, Chungnam, Republic of Korea; 10 Department of Surgery, Research Institute and Hospital, National Cancer Center, Goyang, Republic of Korea; 11 Department of Surgery, Soon Chun Hyang University Hospital, Seoul, Republic of Korea; 12 Division of Breast and Endocrine Surgery, Hallym University Sacred Heart Hospital, Anyang, Republic of Korea; 13 Seoul National University Hospital, Cancer Research Institute, Seoul National University College of Medicine, Seoul, Republic of Korea; 14 Department of Surgery, Gangnam Severance Hospital, Yonsei University College of Medicine, Seoul, Republic of Korea; 15 Department of Surgery, Yeungnam University College of Medicine, Daegu, Republic of Korea; 16 Department of Surgery, Ewha Womans University Mokdong Hospital, Seoul, Republic of Korea; 17 Department of Surgery, Research Institute of Clinical Medicine, Jeonbuk National University Hospital, Jeonbuk National University and Biomedical Research Institute, Jeonju, Republic of Korea; 18 Department of Surgery, Gachon University Gil Hospital, Incheon, Republic of Korea; 19 Department of Internal Medicine, Division of Oncology/Hematology, Korea University College of Medicine, Seoul, Republic of Korea; 20 Department of General Surgery, Busan Paik Hospital, Inje University Surgery, Busan, Republic of Korea; 21 Department of Surgery, Inha University Hospital, Inha University School of Medicine, Incheon, Republic of Korea; 22 Department of Surgery, Soonchunhyang University Bucheon Hospital, Bucheon, Republic of Korea; 23 Department of Surgery, Inje University Sanggye Paik Hospital, Seoul, Republic of Korea; 24 Department of Surgery, Kangbuk Samsung Hospital, Sungkyunkwan University School of Medicine, Seoul, Republic of Korea; 25 Department of Surgery, Myongji Hospital, Goyang, Republic of Korea; 26 Department of Surgery, Konkuk University Medical Center, Konkuk University School of Medicine, Seoul, Republic of Korea; 27 Department of General Surgery, Keimyung University School of Medicine, Daegu, Republic of Korea; 28 Department of Surgery, Daejeon St. Mary’s Hospital, College of Medicine, The Catholic University of Korea, Seoul, Republic of Korea

**Keywords:** breast cancer, premenopausal women, chemotherapy, ovarian function, bone health

## Abstract

**Purpose:**

We compared the impact of tamoxifen alone or with ovarian function suppression (OFS) on bone mineral density (BMD) in premenopausal patients after chemotherapy.

**Methods:**

Of 1483 premenopausal women enrolled in the ASTRRA study, we included 522 who underwent BMD examinations at diagnosis and 3 and 5 years after diagnosis. All BMD measurements were performed using the same scanner in each center across different time points. Patients were stratified into three groups: within the expected range for age (A, Z-score>-1.0), below the expected range (B,-2.0≤ Z-score ≤-1.0), and low bone mineral density for chronological age (C, Z-score< -2.0) groups. We examined changes in groups from baseline to >3-year and 5-year periods to identify any deterioration in BMD. We conducted a subset analysis using the Asan Medical Center (AMC; n=141) data, focusing on the absolute value of bone density (in g/cm^2^ unit).

**Results:**

The 522 included patients (median age, 41.1 years) had a higher bone loss incidence in the OFS addition group at baseline (p=0.028). The tamoxifen-only and tamoxifen+OFS groups did not differ significantly in terms of changes in BMD categories from baseline to 3 (p=0.567) or 5 years (p=0.600). The OFS addition group had a significantly increased risk of BMD deterioration when randomized at the first visit (odds ratio=2.970, p=0.008). Within the AMC subset, the OFS addition group exhibited significantly decreased BMD in the spine (p=0.023) and femur (p=0.040) from the baseline to 3-year period. A non-significantly decreased BMD occurred from the baseline to 5 years in the spine and femur.

**Conclusion:**

Our findings highlighted the deleterious impact on BMD following OFS addition, compared with tamoxifen only treatment. Early OFS exerted an even more detrimental influence on bone health in premenopausal patients with estrogen receptor-positive breast cancer and recovered ovarian function.

**Abbreviations:**

ANOVA, analysis of variance; BMD, one mineral density; CTIBL, Cancer treatment-induced bone loss; DXA, dual-energy X-ray absorptiometry; HER2 human epidermal growth factor receptor 2; L-spin, lumbar spine; OFS, ovarian function suppression; TAM, tamoxifen.

## Introduction

The treatment options of premenopausal patients with estrogen receptor-positive breast cancer is important for breast cancer survivors; however, their treatment remains a considerable challenge. Recent studies, including the ASTRRA (Addition of Ovarian Suppression to Tamoxifen in Young Women With Hormone-Sensitive Breast Cancer Who Remain Premenopausal or Regain Vaginal Bleeding After Chemotherapy) trial, indicate that adding ovarian function suppression (OFS) to tamoxifen (TAM) can yield superior outcomes in premenopausal patients who have undergone adjuvant chemotherapy (1–3). As the prospect of outcomes improves, the side effects associated with OFS addition have received increasing attention to preserve the survivors’ quality of life (2).

The role of bone health in cancer survivor’s quality of life is emerging as an area of significant interest. Decreased bone mineral density (BMD), especially in young females with breast cancer, resulting in bone loss during their life span, can lead to severe complications, such as fractures, severely affecting the quality of life (4, 5). Furthermore, cancer treatment-induced bone loss (CTIBL) is increasingly recognized in young patients with breast cancer undergoing systemic chemotherapy and antihormonal therapy. Although older American Society of Clinical Oncology guidelines have suggested medical intervention for bone health only below specific BMD thresholds, current guidelines advocate for regular bone health monitoring among patients receiving aromatase inhibitors or OFS (6).

Chemotherapy such as taxanes, doxorubicin, cyclophosphamide, and cisplatin are associated with elevated bone resorption and can induce secondary amenorrhea in premenopausal women with breast cancer, resulting in reduced BMD (7, 8). However, studies exploring the impact of hormone therapy, especially regarding the addition of OFS to TAM following chemotherapy, on BMD are relatively rare (7).

The objective of the current study was to fill this gap in knowledge by comparing BMD in premenopausal patients with breast cancer who were taking TAM alone with those taking additional OFS in the ASTTRA trial. Understanding the effects of these treatments on bone health is critical for developing therapeutic strategies that harmonize cancer treatment and preservation of bone health, thereby enhancing the overall quality of life of patients.

## Methods

### Patients

The ASTRRA trial, designed as a phase III, randomized controlled multicenter trial involving 35 institutions across South Korea from March 2009 to March 2014, served as the basis for this study. Briefly, the study included premenopausal patients aged ≤45 years with estrogen receptor-positive, stages I–III breast cancer who had undergone surgical and chemotherapy treatments. Ovarian function was assessed biannually for a span of 2 years by evaluating serum follicle-stimulating hormone levels or any evidence of vaginal bleeding during this period. Once the premenopausal status was confirmed, patients were randomized to complete 5 years of either TAM alone or TAM plus 2 years of OFS (GnRH: Gonadotropin-releasing hormone).

In this retrospective study, we focused on a subset of 522 patients from the ASTRRA cohort with available BMD data categorized at baseline and 3 and 5 years after diagnosis. We further explored the BMD results of the patients enrolled at the Asan Medical Center (AMC) and obtained their specific T-score in units of g/cm^3^.

### Bone health monitoring and BMD assessment

To assess bone health status, BMD was measured at the following intervals: prior to surgery and subsequently 3- and 5-year post-diagnosis. Based on the BMD results, patients were classified into one of three categories: within the expected range for age (A, Z-score>-1.0), below the expected range (B,-2.0≤ Z-score ≤-1.0), and low bone mineral density for chronological age (C, Z-score< -2.0) groups. Patients who transitioned into group B during follow-up were administered calcium and vitamin D supplements and educated on lifestyle modifications to improve bone health. Patients with osteoporosis were asked to consult the endocrinology department for specialized osteoporosis treatment.

BMD was examined using dual-energy X-ray absorptiometry (DXA) with a Hologic QDR densitometer (Hologic, Inc., Waltham, MA). All BMD measurements were performed using the same scanner at each center across different time points. The lumbar spine (L-spine), femoral neck, and total femoral area on the right side were assessed, and the L-spine and total femur data were analyzed.

### Statistical analysis

To investigate the baseline characteristics, patients were stratified into two groups: those who received TAM and those who received TAM combined with OFS. To assess the significance of the differences between groups, two-sided chi-squared analysis and Fisher’s exact test were utilized. Changes in BMD according to each treatment were examined using the t-test, whereas analysis of variance (ANOVA) was used to analyze differences among the treatment groups at baseline and 3- and 5-year intervals.

All statistical tests were two-tailed, and a p-value <0.05 was considered statistically significant. Statistical analyses were performed using the IBM SPSS Statistics for Windows, ver. 20 (IBM Corp., Armonk, NY, USA).

## Results

### Patient characteristics

**Figure 1:**
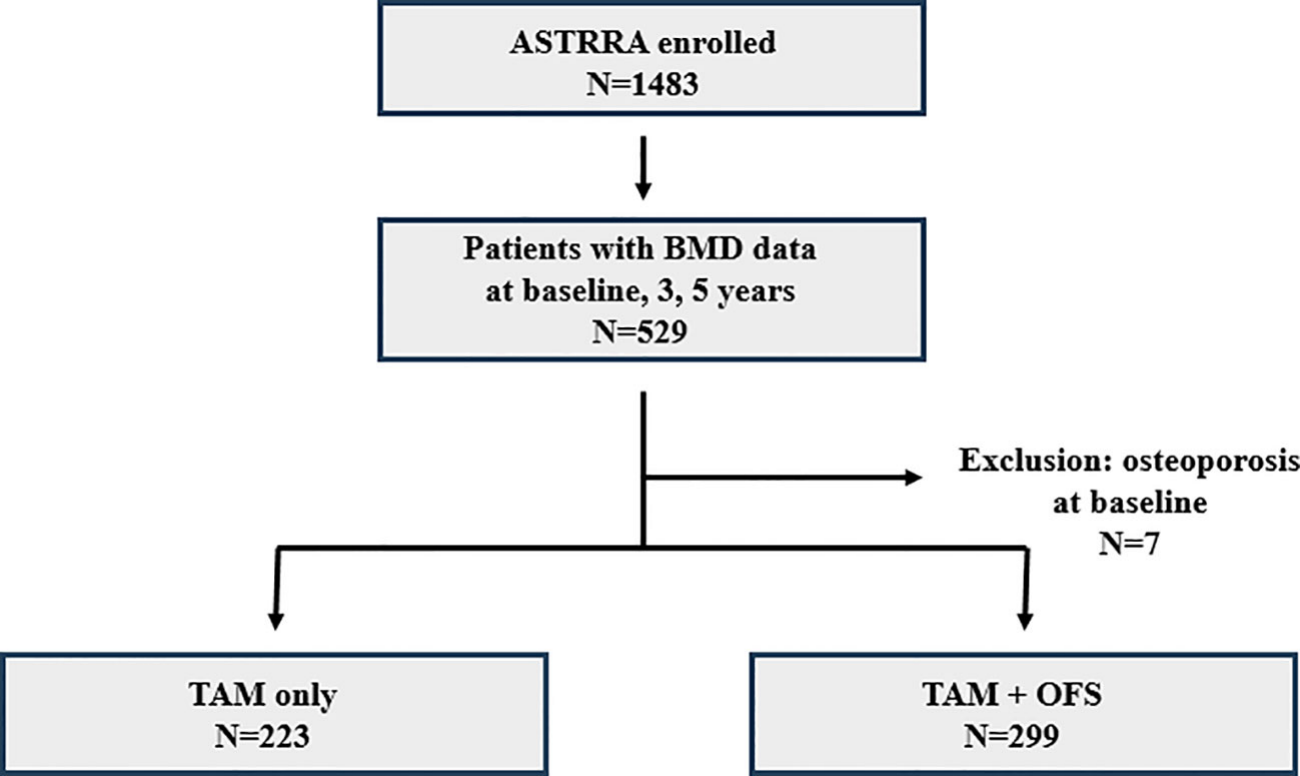
Flowchart showing patients included this study.

The study cohort comprised 522 patients from the ASTRRA trial whose BMD measurements were collected for at least 5 years. Among the 522 selected patients, 223 (42.72%) and 229 (57.28%) were assigned to the TAM-only and TAM+OFS groups, respectively (**Figure 1**). No statistically significant differences were observed between the two groups in terms of baseline characteristics, including age, lymph node status, tumor size, tumor grade, histology, human epidermal growth factor receptor 2 (HER2) status, chemotherapy regimen, and surgery or radiotherapy history, showing comparable cohorts after randomization (**Table 1**).

**Table 1:** Patient characteristics.

	TAM only group (n=223)(%)	TAM+OFS group (n=299)(%)	*p*-value
Age at enrollment, years			0.199
<35	25 (11.21)	36 (12.04)	
35-39	67 (30.04)	69 (23.08)	
40-45	131 (58.74)	194 (64.88)	
Lymph node status			0.764
Negative	97 (43.5)	134 (44.82)	
Positive	126 (56.5)	165 (55.18)	
Tumor size			0.851
<2cm	113 (50.67)	154 (51.51)	
≥2cm	110 (49.33)	145 (48.49)	
Tumor grade			0.781
G1	36 (16.14)	58 (19.4)	
G2	113 (50.67)	150 (50.17)	
G3	52 (23.32)	63 (21.07)	
Unknown	22 (9.87)	28 (9.36)	
Histology			0.833
IDC	201 (90.13)	266 (88.96)	
ILC	9 (4.04)	12 (4.01)	
Other	13 (5.83)	19 (6.35)	
Unknown	0 (0)	2 (0.67)	
HER2 status			0.326
Negative	122 (54.71)	183 (61.2)	
Positive	27 (12.11)	30 (10.03)	
Unknown	74 (33.18)	86 (28.76)	
Chemotherapy regimen			0.127
AC	63 (28.25)	93 (31.1)	
ACT	124 (55.61)	161 (53.85)	
AT	7 (3.14)	12 (4.01)	
FAC	17 (7.62)	29 (9.7)	
Other	2 (0.9)	2 (0.67)	
TAC	7 (3.14)	2 (0.67)	
Unknown	3 (1.35)	0 (0)	
Surgery			0.535
TM	74 (33.18)	111 (37.12)	
BCS	136 (60.99)	175 (58.53)	
Unknown	13 (5.83)	13 (4.35)	
Radiotherapy			0.756
Done	138 (61.88)	189 (63.21)	
Not done	85 (38.12)	110 (36.79)	

### Changes in BMD

**Table 2** illustrates that the temporal changes in BMD between the two groups showed distinct trends; in the table, ‘change’ refers to the transition from one category to another. At baseline, the TAM-only group had a significantly higher percentage of patients with normal (Z-score>-1.0) (82.06%) than that of the TAM+OFS group (73.91%; p=0.028). However, this difference narrowed over time, especially at the 5-year follow-up, but did not reach a statistical significance level (p=0.058). In both groups, patients gradually experienced a progressive decline in bone density, with no significant between-group differences (**Table 2**).

**Table 2:** Temporal variation of BMD status and changes in BMD overtime intervals.

		TAM only group	TAM+OFS group	*p*-value
		(N = 223)(%)	(N = 299)(%)	
BMD				
baseline	Group A	183 (82.06)	221 (73.91)	0.028
	Group B	40 (17.94)	78 (26.09)	
3yr	Group A	119 (57.49)	149 (54.58)	0.793
	Group B	85 (41.06)	119 (43.59)	
	Group C	3 (1.45)	5 (1.83)	
5yr	Group A	130 (58.3)	156 (52.17)	0.058
	Group B	84 (37.67)	138 (46.15)	
	Group C	9 (4.04)	5 (1.67)	
Change				
3yr-baseline	No change or better	153 (73.91)	208 (76.19)	0.567
	worse	54 (26.09)	65 (23.81)	
5yr-baseline	No change or better	161 (72.2)	222 (74.25)	0.600
	worse	62 (27.8)	77 (25.75)	
5yr-3yr	No change or better	189 (91.3)	248 (90.84)	0.861
	worse	18 (8.7)	25 (9.16)	

**Figure 2:**
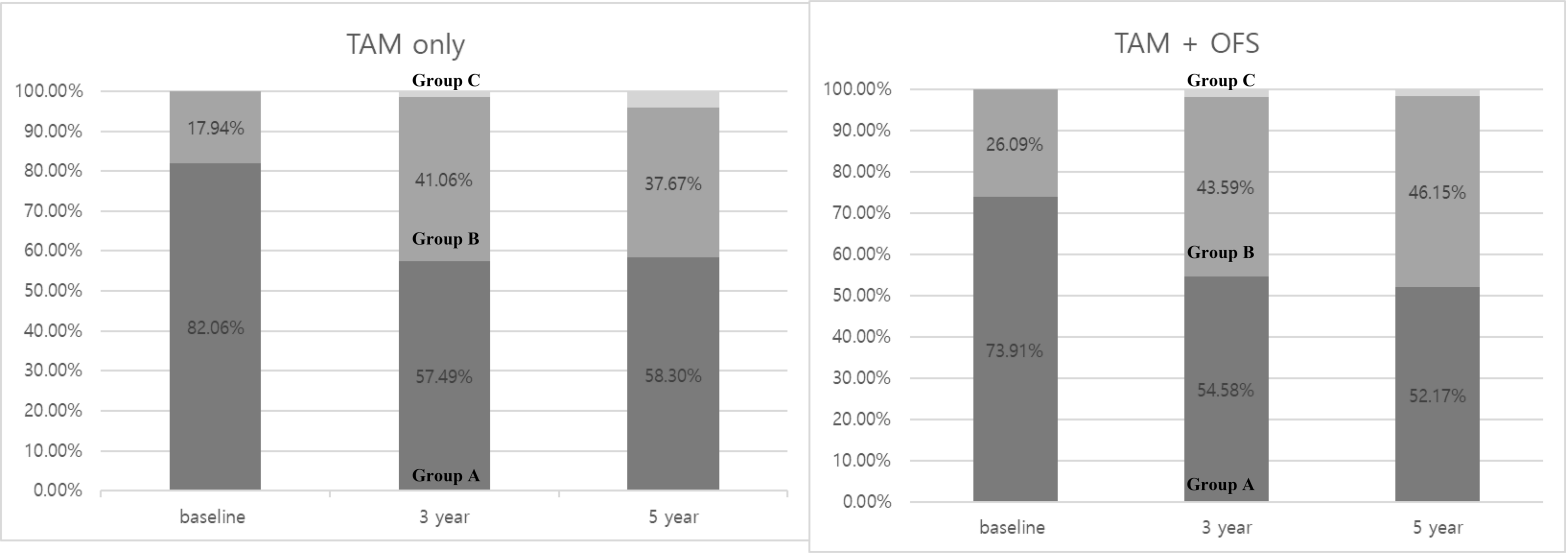
Proportions of BMD group in two groups.

Interestingly, the proportion of patients with normal BMD was substantially reduced in both treatment groups during the initial 3-year period, declining from 82.06 to 57.49% in the TAM-only group and from 73.91 to 54.58% in the TAM+OFS group (**Figure 2**).

**Figure 3:**
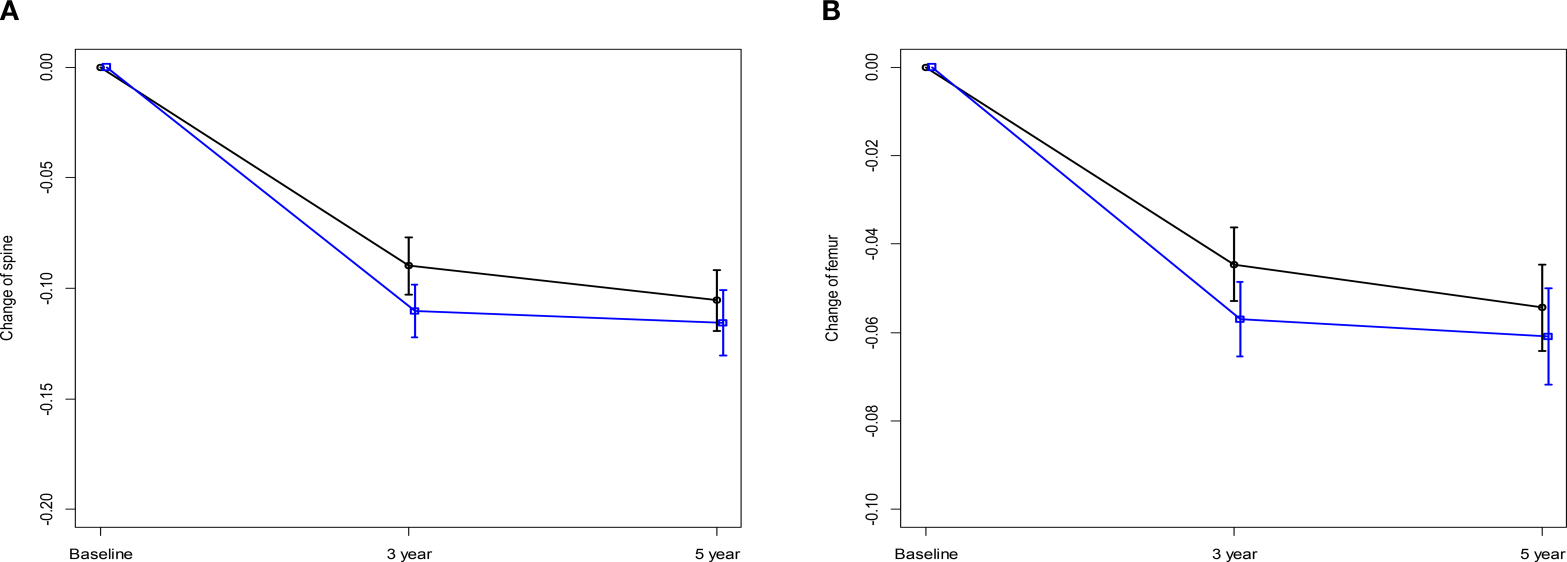
Changes in Z-score over 5 years **(A)** Change of spine. **(B)** Change of femur, black line: TAM only, blue line: TAM+OFS.

In a subgroup analysis focusing on patients from the AMC cohort, fluctuations in T-scores were observed for the spine and femoral regions. For most time intervals examined, no significant differences were reported between the TAM-only and TAM+OFS groups. However, we noted a significant divergence in T-scores between the two treatment groups over the baseline to 3-year period (spine: p=0.023, femur: p=0.040). However, this significance level was not sustained in the subsequent 2-year period (**Tables 3**, **4**; **Figure 3**).

**Table 3:** Temporal variation of Z-score and changes in Z-score over time intervals within the AMC cohort.

	Time interval(months)	TAM only (ref)(%)	TAM+OFS(%)	Odds Ratio(95% CI)	*p*-value
Overall		62/223(27.8)	77/299(25.75)	0.91(0.61-1.33)	0.008
Visit1	0	4/31(12.9)	11/36(30.56)	2.97(0.84-10.55)	
Visit2	6	40/116(34.48)	39/158(24.68)	0.62(0.37-1.05)	
Visit3	12	13/42(30.95)	16/70(22.86)	0.66(0.28-1.56)	
Visit4	18	5/21(23.81)	7/23(30.43)	1.40(0.37-5.35)	
Visit5	24	0/13(0)	4/12(33.33)	Infinity	

**Table 4:** Variations in BMD from baseline to 5-years stratified by randomization time intervals.

	TAM only(n=73)	TAM + OFS(n=68)	*p*-value
Spine (AP)			
baseline	1.14 ± 0.12	1.16 ± 0.14	0.337
3yr	1.05 ± 0.12	1.05 ± 0.13	0.990
5yr	1.03 ± 0.12	1.04 ± 0.14	0.638
Change			
3yr-baseline	0.09 ± 0.06	-0.11 ± 0.05	0.023
5yr-baseline	-0.11 ± 0.06	-0.12 ± 0.06	0.324
5yr-3yr	-0.02 ± 0.04	-0.01 ± 0.04	0.158
Femur			
baseline	0.98 ± 0.11	0.97 ± 0.12	0.678
3yr	0.94 ± 0.11	0.93 ± 0.12	0.688
5yr	0.93 ± 0.11	0.92 ± 0.12	0.839
Change			
5yr-baseline	-0.05 ± 0.04	-0.06 ± 0.04	0.375
5yr-baseline	-0.05 ± 0.04	-0.06 ± 0.04	0.375
5yr-3yr	-0.01 ± 0.03	0 ± 0.03	0.124

### Variations in BMD change according to the randomization period

Herein, we performed an in-depth analysis of BMD variations from baseline to 5 years, particularly focusing on the influence of randomization timing. After randomization at the initial visit after enrollment, 12.9 and 30.56% of patients were assigned to the TAM-only and TAM+OFS groups, respectively. Additionally, patients were randomized during their second (TAM-only: 34.48%, TAM+OFS: 22.86%) and third (TAM-only: 30.95%, TAM+OFS: 22.86%) visits post-enrollment. Notably, considering patients who were randomized at the first visit, there was a significantly increased risk of experiencing BMD changes when compared with those randomized at later visits (odds ratio [OR] = 2.97, 95% confidence interval [CI] = 0.84 – 10.55, p=0.008).

## Discussion

Herein, we found no significant differences in the overall rate of BMD change between the two treatment groups over 3- and 5-year follow-ups when patients were categorized into within the expected range for age group (A, Z-score>-1.0), below the expected range group (B,-2.0≤ Z-score ≤-1.0), and low bone mineral density for chronological age group (C, Z-score< -2.0) groups. However, the proportions of patients categorized as having normal BMD substantially declined during the initial 3-year period in both treatment groups. Furthermore, a detailed analysis of T-score values revealed significant differences in the initial 3-year interval, indicating a greater decrease in T-scores in the TAM+OFS group compared with these in the TAM-only group. The timing of randomization emerged as an important factor influencing BMD outcomes. Specifically, patients who were randomized to start OFS at the initial visit after enrollment exhibited a significantly increased risk of experiencing BMD changes. This finding emphasizes the importance of timing when initiating hormonal therapies that include OFS, as early initiation appears to detrimentally impact bone health (8).

Historically, research on BMD has predominantly focused on postmenopausal women, given that postmenopausal status is a critical factor in bone loss. These studies have primarily explored the hormonal effects on bone health. However, with the advent of diverse antihormonal treatments, including different regimens and periods, the impact of these antihormonal interventions on bone health in premenopausal women needs to be established.

Initially, when discussing the etiological factors of bone loss during breast cancer treatment, potential factors include chemotherapy, tamoxifen, and OFS. Chemotherapy, especially cyclophosphamide, has been associated with bone density decrease (9). The chemotherapy-mediated deterioration of ovarian function has been identified as an instigator of bone loss (10). However, some studies suggest that post-chemotherapy bone loss is not merely a result of estrogen depletion but rather associated with the cytotoxic impact of chemotherapy on bones (11, 12). Consequently, a hypothesis has linked antihormonal therapy to changes in BMD, although its implications remain inclusive (13). Moreover, the ambivalent effect of TAM on bone health has been suggested. Vehmanen et al. (11) reported that post-chemotherapy, when menstruation resumes, TAM functions as an estrogen antagonist. Conversely, under conditions of amenorrhea, TAM exerts opposite functions, providing a protective benefit to bone density (14, 15). Predominantly, in premenopausal women, bone density declines concurrently with antihormonal treatment (16). Kim et al. (17) revealed that the effect of TAM plus OFS causes a comparable degree of bone loss to that induced by chemotherapy. Particularly in groups where OFS was added, cortical porosity and trabecular deterioration were reportedly associated with estradiol depletion (4). Additionally, in another study, after 2 years of OFS administration, its effects were found to be reversible, impacting both ovarian function and bone health (5, 13). Based on this evidence, TAM and OFS may reduce BMD through distinct mechanisms in patients experiencing ovarian function recovery. Furthermore, considering the reversible nature of these effects, initiating OFS immediately after the recovery of ovarian function post-adjuvant chemotherapy may have substantial implications on the bone health of premenopausal women.

The superiority of OFS addition to TAM post-chemotherapy, as suggested by the ASTRRA and SOFT-TEXT trials, could improve therapeutic outcomes (3, 18, 19). However, reduced bone health, a concern evident from our baseline findings, revealed that a higher incidence of bone loss was observed in the OFS addition group. Regarding the management of bone health, monitoring and treatment practices, such as BMD testing and administration of antiresorptive agents, are mainly conducted among postmenopausal women owing to the prevalent incidence of osteoporosis within the demographic. Conversely, there is minimal focus on younger patients despite these patients undergoing chemotherapy and antihormonal therapy, both of which are related to bone health. This is of clinical significance, especially considering that diminished bone density in younger patients can culminate into osteopenia or osteoporosis, compromising their quality of life in the long run owing to potential complications such as fractures. Furthermore, close collaboration with policymakers is essential to ensure the effective implementation of such medical practices.

Regarding the treatment for reduced BMD, the primary modalities include calcium carbonate/cholecalciferol and antiresorptive agents, such as denosumab and bisphosphonates. Traditionally, antiresorptive agents have been prescribed to postmenopausal women, partly owing to under-monitoring and the rarity of early intervention in young patients. Antiresorptive agents function by inhibiting the release of calcium ions from bone and are typically administered based on a T-score of less than -2.5 (20). Certain studies, including the HOBOE trial, have suggested that these agents exert benefits beyond bone health, improving breast cancer outcomes (21). According to the HOBOE trial, the combination of bisphosphonate and ovarian suppression enhances disease-free survival in premenopausal patients, accompanied by an increase in toxicity when compared with patients who did not receive bisphosphonates. Additionally, a review study found that early intervention with antiresorptive agents, such as denosumab and bisphosphonates, could positively impact disease recurrence, locoregional recurrence, and resistance to secondary endocrine therapy rather than primary resistance (22). However, due to the risk of rebound bone loss and associated vertebral fractures following discontinuation of denosumab, many endocrinology experts are hesitant to use. Consequently, bisphosphonates are currently preferred, especially in young patients. Supported by these findings, early assessment and proactive management of bone health, including appropriate diagnostic evaluation and pharmacologic intervention in premenopausal women, is valuable and should be integrated into clinical practice.

Regarding the limitations of the current study, this was a retrospective cohort analysis from the ASTRRA trial, and the primary focus of the ASTRRA study was to assess the cancer outcomes associated with the addition of OFS to TAM. Accordingly, there was a lack of etiological data, including body mass index, hormonal profiles, and lifestyle behaviors, all of which could influence BMD. Moreover, the trial lacked comparative cohorts, such as subjects who received only chemotherapy or no intervention. Therefore, if matching patients with control groups is possible, it would enhance the precision of the analysis.

Nevertheless, the current study has substantial implications for the clinical management of breast cancer, particularly in relation to the bone health of young patients with breast cancer. For premenopausal women experiencing the resumption of ovarian function post-chemotherapy, this study advocates for a more careful approach to BMD monitoring and early intervention if needed (23). This requires a comprehensive consideration extending beyond bone health to long-term quality of life consequences (24). As the importance of bone health in the quality of life of cancer survivors’ gains increasing attention, the need for further large-scale randomized controlled trials to explore the effects of various antihormonal therapy combinations on bone health becomes imperative to formulate evidence-based clinical guidelines.

## Conclusion

Collectively, this study identified that the addition of OFS to TAM adversely impacts BMD. Notably, early initiation of OFS accentuates the negative impact on bone health, especially in premenopausal patients with estrogen receptor-positive breast cancer who have regained ovarian function.

## Data Availability

The data analyzed in this study is subject to the following licenses/restrictions: The datasets analyzed during the current study are not publicly available as the personal information of patients must be protected but are available from the corresponding author on reasonable request. Requests to access these datasets should be directed to Hee Jung Kim, heejeongkim.br@gmail.com.
