# Correction: Comparative study on bone mineral density in premenopausal patients with estrogen receptor-positive breast cancer in ASTRRA Study: a 5-year follow-up study

**DOI:** 10.3389/fonc.2025.1729229

**Published:** 2025-11-07

**Authors:** Eunju Shin, Seung Il Kim, Min-ho Park, Hyun-Ah Kim, Yongsik Jung, Jai Min Ryu, Eun Hwa Park, Sung Yong Kim, Eun-Gyeong Lee, Min Hyuk Lee, Jung Ho Park, Seock-Ah Im, Soong June Bae, Su Hwan Kang, Woo Sung Lim, Hyun Jo Youn, Heung Kyu Park, Kyong Hwa Park, Tae Hyun Kim, Shin Young Park, Cheol Wan Lim, Geum Hee Kwak, Chanheun Park, Hyuk Jae Shin, Young Bum Yoo, Sun Hee Kang, Bong Kyun Kim, Hee Jeong Kim

**Affiliations:** 1 Division of Breast Surgery, Department of Surgery, University of Ulsan College of Medicine, Asan Medical Center, Seoul, Republic of Korea; 2 Department of Surgery, Kyung Hee University Hospital at Gangdong, Seoul, Republic of Korea; 3 Department of Surgery, Yonsei University College of Medicine, Seoul, Republic of Korea; 4 Department of Surgery, Chonnam National University Medical School, Chonnam National University Hwasun Hospital, Jeollanam-do, Republic of Korea; 5 Department of Surgery, Korea Cancer Center Hospital, Korea Institute of Radiological and Medical Sciences, Seoul, Republic of Korea; 6 Department of Surgery, Ajou University School of Medicine, Suwon, Republic of Korea; 7 Division of Breast Surgery, Department of Surgery, Samsung Medical Center, Sungkyunkwan University School of Medicine, Seoul, Republic of Korea; 8 Department of Surgery, Dong-A University College of Medicine, Busan, Republic of Korea; 9 Department of Surgery, Soonchunhyang University, College of Medicine, Cheonan Hospital, Chungnam, Republic of Korea; 10 Department of Surgery, Research Institute and Hospital, National Cancer Center, Goyang, Republic of Korea; 11 Department of Surgery, Soon Chun Hyang University Hospital, Seoul, Republic of Korea; 12 Division of Breast and Endocrine Surgery, Hallym University Sacred Heart Hospital, Anyang, Republic of Korea; 13 Seoul National University Hospital, Cancer Research Institute, Seoul National University College of Medicine, Seoul, Republic of Korea; 14 Department of Surgery, Gangnam Severance Hospital, Yonsei University College of Medicine, Seoul, Republic of Korea; 15 Department of Surgery, Yeungnam University College of Medicine, Daegu, Republic of Korea; 16 Department of Surgery, Ewha Womans University Mokdong Hospital, Seoul, Republic of Korea; 17 Department of Surgery, Research Institute of Clinical Medicine, Jeonbuk National University Hospital, Jeonbuk National University and Biomedical Research Institute, Jeonju, Republic of Korea; 18 Department of Surgery, Gachon University Gil Hospital, Incheon, Republic of Korea; 19 Department of Internal Medicine, Division of Oncology/Hematology, Korea University College of Medicine, Seoul, Republic of Korea; 20 Department of General Surgery, Busan Paik Hospital, Inje University Surgery, Busan, Republic of Korea; 21 Department of Surgery, Inha University Hospital, Inha University School of Medicine, Incheon, Republic of Korea; 22 Department of Surgery, Soonchunhyang University Bucheon Hospital, Bucheon, Republic of Korea; 23 Department of Surgery, Inje University Sanggye Paik Hospital, Seoul, Republic of Korea; 24 Department of Surgery, Kangbuk Samsung Hospital, Sungkyunkwan University School of Medicine, Seoul, Republic of Korea; 25 Department of Surgery, Myongji Hospital, Goyang, Republic of Korea; 26 Department of Surgery, Konkuk University Medical Center, Konkuk University School of Medicine, Seoul, Republic of Korea; 27 Department of General Surgery, Keimyung University School of Medicine, Daegu, Republic of Korea; 28 Department of Surgery, Daejeon St. Mary’s Hospital, College of Medicine, The Catholic University of Korea, Seoul, Republic of Korea

**Keywords:** breast cancer, premenopausal women, chemotherapy, ovarian function, bone health

An incorrect **Funding** statement was provided. The correct **Funding** statement reads:

“This research was supported by a grant of the Patient-Centered Clinical Research Coordinating Center (PACEN) funded by Ministry of Health & Welfare, Republic of Korea (grant number: RS-2024-00396822) and the Asan Institute for Life Sciences, Asan Medical Center, Seoul, Korea (grant number: 2020IF0004).”

The original version of this article has been updated.

